# Oxygen and pH-sensitivity of human osteoarthritic chondrocytes in 3-D alginate bead culture system

**DOI:** 10.1016/j.joca.2013.06.028

**Published:** 2013-11

**Authors:** J.A. Collins, R.J. Moots, R. Winstanley, P.D. Clegg, P.I. Milner

**Affiliations:** † Department of Musculoskeletal Biology, Institute of Ageing and Chronic Disease, University of Liverpool, Leahurst Campus, Neston, Cheshire CH64 7TE, UK; ‡ Department of Musculoskeletal Biology, Institute of Ageing and Chronic Disease, University of Liverpool, University Hospital, Aintree, Liverpool L9 7AL, UK

**Keywords:** Hypoxia, pH, Redox imbalance, Mitochondria, Osteoarthritis

## Abstract

**Objective:**

To identify the effect of alterations in physical parameters such as oxygen and pH on processes associated with cellular redox balance in osteoarthritic chondrocytes.

**Method:**

Human osteoarthritic chondrocytes (HOAC) were isolated from total knee arthroplasty samples and cultured in 3-D alginate beads in four different oxygen tensions (<1%, 2%, 5% and 21% O_2_), at pH 7.2 and 6.2 and in the presence or absence of 10 ng/ml, interleukin-1β (IL-1β). Cell viability, media glycosaminoglycan (GAG) levels, media nitrate/nitrate levels, active matrix metalloproteinase (MMP)-13 and intracellular adenosine triphosphate (ATP_i_) were measured over a 96-h time course. Intracellular reactive oxygen species (ROS), mitochondrial membrane potential, intracellular pH and reduced/oxidised glutathione (GSH/GSSG) were additionally measured after 48-h incubation under these experimental conditions.

**Results:**

Hypoxia (2% O_2_) and anoxia (<1% O_2_), acidosis (pH 6.2) and 10 ng/ml IL-1β reduced HOAC cell viability and increased GAG media levels. Acidosis and IL-1β increased nitrite/nitrate release, but increases were moderate at 2% O_2_ and significantly reduced at <1% O_2_. ATP_i_ was significantly reduced following hypoxia and anoxia and acidosis. At 48 h cellular ROS levels were increased by acidosis and IL-1β but reduced in hypoxia and anoxia. Mitochondrial membrane potential was reduced in low oxygen, acidosis and IL-1β. Anoxia also resulted in intracellular acidosis. GSH/GSSG ratio was reduced in low oxygen conditions, acidosis and IL-1β.

**Conclusions:**

This study shows that oxygen and pH affect elements of the redox system in HOAC including cellular anti-oxidants, mitochondrial membrane potential and ROS levels.

## Introduction

Articular cartilage is a highly specialised tissue designed to allow pain-free friction-less movement across joints^1^. Mature cartilage is avascular, relatively hypoxic and acidic and thus provides an unusual and challenging environment to the resident cell, the chondrocyte^2^. The avascular nature of cartilage results in chondrocytes exposed to relatively low concentrations of oxygen compared to other tissue types, with diffusion gradients existing through cartilage from around 12% at the surface, to around 2% in the deeper layers^3^. In addition to this hypoxic environment, articular chondrocytes are embedded within an extracellular matrix rich in collagen type II and proteoglycans. The fixed negatively charged proteoglycans are responsible for the compressive stiffness of articular cartilage, by attracting free mobile cations (e.g., Na^+^, H^+^) and osmotically obliged water, resulting in a hydrated matrix of increased osmolarity and reduced pH^4^. As well as the acidic extracellular environment, production of lactate from predominately anaerobic glycolytic metabolism leads to increased intracellular acid loads experienced by chondrocytes.

In joint diseases, such as osteoarthritis and rheumatoid arthritis, oxygen levels are reduced further from increased consumption by inflammatory cells, such as synoviocytes, and reduced delivery of oxygen to synovial fluid due to joint capsule fibrosis and subchondral bone sclerosis^5,6^. Alteration in the physical environment and release of inflammatory mediators by articular cells (under disease conditions) leads to acidosis^7–9^. Since matrix synthesis and activity of many degradatory enzymes are oxygen and pH-sensitive^10,11^, changes in the physiochemical environment of the chondrocyte are likely to have important effects on chondrocyte function and hence cartilage integrity. Attenuation of the mechanisms involved in intracellular pH regulation has been previously demonstrated under short-term hypoxic conditions^12,13^.

Components of cellular redox balance have been shown to have oxygen and pH-sensitivity in other tissues^14,15^, and redox imbalance has been shown to be a feature of osteoarthritis^16,17^. How extracellular factors such as hypoxia and acidosis affect cellular redox mechanisms in articular chondrocytes is unknown but likely to have implications for disease progression and potential therapeutic intervention. This study investigated the effect of changes to oxygen tension and pH and exposure to the pro-inflammatory cytokine, interleukin (IL)-1β, on cellular oxidant systems and mitochondrial function in human osteoarthritic chondrocytes (HOAC) cultured in 3-D alginate beads.

## Method

### Chondrocyte isolation and cell culture

Human articular chondrocytes were isolated from total knee arthroplasty samples (total number of donors, *n* = 9; mean age of donors: 71.8 years) with informed consent and ethical approval (in accordance with the Cheshire Research Ethics Committee). Full thickness macroscopically intact cartilage samples were taken and mixed randomly for each individual. Cartilage slices were suspended in Dulbecco's Modified Eagle's Medium (DMEM) (glucose 1,000 mg/l) minus Phenol Red and sodium bicarbonate (D2902, Sigma Aldrich, Dorset, UK), supplemented with 10% foetal calf serum (FCS), 100 units/ml penicillin, 100 μg/ml streptomycin and 500 ng/ml amphotericin B and buffered with 25 mM 4-(2-hydroxyethyl)-1-piperazineethanesulfonic acid (HEPES). Chondrocytes were liberated from cartilage matrix by standard enzymatic digestion protocols (collagenase type II 0.8 mg/ml, 37°C, 21% O_2_ 15 h).

For 96-h time course experiments, primary chondrocytes were expanded in monolayer culture to achieve adequate cell number prior to 3-D alginate bead culture. For experiments where 48-h time point only was examined, isolated primary chondrocytes (P0) were cultured directly in alginate beads. For monolayer expansion, chondrocytes were seeded onto 175 cm^2^ tissue culture flasks at cell densities of 2 × 10^3^ cells/cm^2^ and grown to confluence at 37°C in humidified 5% O_2_/5% CO_2_ (balanced with 90% N_2_). At P3, chondrocytes were harvested from monolayer expansion and re-suspended in low viscosity (1.2%) alginate solution at a cell density of 4 × 10^6^ cell/ml. The alginate/cell suspension was released dropwise into Petri dishes containing calcium chloride (102 mM). After instantaneous gelation, beads were left to polymerise. Following polymerisation, beads were washed twice in NaCl (150 mM) and incubated in DMEM (minus Phenol Red and sodium bicarbonate plus 10% FCS with 25 mM HEPES, pH 7.2) for 14 days at 5% O_2_ with media changes every 48 h. Fourteen days is a recommended period of time to allow cells to maintain chondrocyte activity^17,18^. However, to assess the phenotype of the cells at this stage, aliquots were harvested prior to the 96-h experimental time course. For this, cells were released from the alginate matrix by depolymerisation in 55 mM sodium citrate solution (10 min, 37°C) and centrifuged with cell pellets re-suspended in sodium dodecyl sulfate (SDS) loading buffer and the cell lysate sonicated before storage at −80°C for COL2A1, cartilage oligomeric matrix protein (COMP) and SOX9 expression.

Remaining beads were incubated in DMEM (minus Phenol Red and sodium bicarbonate plus 10% FCS) with 25 mM HEPES (pH 7.2) or 10 mM piperazine-N,N′-bis(2-ethanesulfonic acid) (PIPES) (pH 6.2) in the presence or absence of IL-1β (10 ng/ml) for 24, 48 or 96 h at <1%, 2%, 5% or 21% O_2_. Media were equilibrated at appropriate oxygen tensions (<1, 2, 5% and 21%) prior to the start of this incubation period, verified by an oxygen electrode (Oxyview-1, Hanstech, Norfolk, UK). For <1% O_2_, sodium dithionite (1.5 mM) was added to the media to scavenge free available oxygen.

Following 24, 48 or 96 h incubation, cells were released from their alginate matrix. Liberated chondrocytes were centrifuged at 1,400 rpm and washed in sterile phosphate buffered saline (PBS) (equilibrated to required oxygen tension). Cell viability was assessed using the Trypan blue exclusion method. Aliquots of cells were also taken following 96-h experimental period to assess articular chondrocyte phenotype again. O_2_ and pH measurements of the media were made and the remaining media sample frozen (−20°C) for later analysis.

### COL2A1, COMP and SOX9 analysis

Total protein from cell lysates was calculated using the bicinchoninic acid (BCA) protein quantification assay. Cell lysates (20 μg, *n* = 3) were electrophoresed and separated on 4–12% sodium dodecyl sulphate–polyacrylamide gel electrophoresis (SDS-PAGE) gels (Nu-Page, Life Technologies, Paisley, UK) before transfer to a nitrocellulose membrane. Membranes were blocked using a PBS/Tween buffer and non-fat dried milk (w/v 1%) solution for 1 h followed by overnight incubation (15 h, 4°C) with primary antibodies against collagen type II (COL2A1, 1:5,000 dilution) (sc-7764, Santa Cruz Biotechnology, Heidelberg, Germany), COMP (1:1,000 dilution) (*kind gift from Dr J Dudhia*) and SOX9 (1:500 dilution) (AB5535 Millipore, Watford, UK), to detect for markers of a chondrocyte phenotype with α-Tubulin (1:1,000 dilution) (ab4074, Abcam, Cambridge, UK) as loading control. Membranes were washed and incubated in a secondary horseradish peroxidase conjugated antibody (1:2,000 dilution). Antigen-antibody complexes were viewed by enhanced chemiluminescence inside a Ultra-Violet Products Ltd (UVP, Cambridge, UK) ChemiDoc-it imaging system. Blots were imaged using VisionWorksLS image acquisition software package and band densities were analysed using ImageJ 1.42. Results were normalised to the loading control.

### Glycosaminoglycan (GAG) release

Dimethylmethylene blue (DMMB) assay was used to assess the concentration of sulphated GAG in media samples^19^. A standard curve was produced using chondroitin sulphate C as a standard. 40 μl of each standard and 40 μl from each spent media sample were entered into a 96 well plate. From a stock solution of DMMB dye 250 μl was entered into each unknown sample and standard. Absorbance was immediately read on a spectrophotometer at absorbance of 570 nm. GAG concentrations from media samples were calculated using the standard curve.

### Intracellular ATP (ATP_i_) measurement

ATP_i_ levels were quantified using the EnzyLight^™^ ATP Assay Kit (BioAssay Systems, Hayward, CA, USA) as specified by the manufacturer's instructions. A standard curve was produced using ATP standards. 10 μl of each standard and 10 μl cell suspension were added to each well in a 96 well plate with 90 μl Reconstitution Reagent (containing assay buffer, substrate and ATP enzyme (95:1:1)). Luminescence was immediately read on a Bio-Tek FLX800 luminescent plate reader and ATP_i_ calculated from the standard curve.

### Nitric oxide (NO) measurement in media

NO production was determined by measuring the stable metabolic end products of NO release (nitrite and nitrate) in culture media using the Griess reaction^20^. Nitrite and nitrite plus nitrate calibration curves were created and nitrite and nitrate content calculated from samples by measuring absorbance at 550 nm.

### Active MMP-13 enzyme-linked immunosorbent assay (ELISA)

Active matrix metalloproteinase (MMP)-13 present in media was quantified using the MMP-13 Human ELISA Kit (Abcam, Cambridge, UK) as per manufacturer's instructions.

### Reactive oxygen species (ROS)

ROS levels were measured using the dichlorofluorescin (DCF) method^21^. After incubation at relevant oxygen tension, liberated chondrocytes were loaded with 10 μM DCF-DA for 40 min (<1, 2, 5 or 21% O_2_, 37°C) before washing twice in PBS (equilibrated at relevant O_2_ tension) and DCF fluorescence measured spectrophotometrically (excitation: 490 nm/emission 535 nm).

### Mitochondrial membrane potential (ΔΨ_m_)

Mitochondrial membrane potential was measured using 5,5′,6,6′-tetrachloro-1,1′,3,3′-tetraethylbenzimidazolylcarbocyanine iodide (JC-1)^22^. Cells were loaded with 50 μg/ml JC-1 for 40 min (<1, 2, 5 or 21% O_2_, 37°C) then washed twice in PBS (equilibrated at relevant O_2_ tension) before 5 μl aliquots of cell suspension were fixed on cover slides (Vectashield Mounting Medium). Intensity of red and green fluorescence emission was measured (excitation: 490 nm, emission 585 nm for red and 514 nm for green) using Nikon Eclipse TS-100 inverted microscope with Eclipse TS-100/TS-100-F Epi-fluorescence attachment and red:green intensity ratio calculated.

### Intracellular pH measurement

The ratiometric dye 2′, 7′-bis-(2-carboxyethyl)-5-(and-6)-carboxyfluorescein) (BCECF) was used to measure intracellular pH (pH_i_). Chondrocytes were loaded with 10 μM BCECF-AM for 30 min (<1, 2, 5 or 21% O_2_, 37°C) before washing twice in PBS (equilibrated at relevant O_2_ tension) and BCECF fluorescence measured spectrophotometrically (dual excitation: 435 and 490 nm/emission 535 nm). The high K^+^-nigericin method was used to create a pH-calibration curve^12^. Briefly, separate aliquots of cells were loaded with 10 μM BCECF-AM for 30 min (37°C) then washed in PBS and re-suspended in high K^+^ solutions containing the ionophore nigericin (2 μM) of differing external pH before measuring BCECF fluorescence.

### GSH:GSSG

Cellular GSH:GSSG ratio was determined by the GSH/GSSG-Glo™ Assay. Total glutathione and oxidised glutathione (GSSG) were assayed as specified by the manufacturer's instructions. From total glutathione and oxidised glutathione, reduced glutathione (GSH) was determined and from this GSH/GSSG ratio calculated.

### Statistical analysis

Unless otherwise stated, data are presented as mean ± 95% confidence intervals (CI) where one knee was used from one individual and each *n* number for each parameter measured was performed in triplicate. Data were tested for normality using the Kolmogorov–Smirnov test. Significant differences were determined by one-way analysis of variance (ANOVA) with Tukey and Dunnett's *post-hoc* corrections (SPSS Inc. Chicago IL, USA). A *P*-value of <0.05 was considered statistically significant. Unless otherwise stated, results are presented as % control (5% O_2_, pH 7.2, time = 0).

## Results

### Chondrocyte phenotype, viability and media conditions

Western blot analysis showed no change in COL2A1, COMP or SOX9 expression following culture period in alginate beads, indicating no detectable alterations to articular chondrocyte phenotype^23^ during the study (Supplementary material, Fig. S1).

Media pH remained constant over 96 h in all conditions apart from <1% O_2_, where reductions occurred within 24 h to values around pH 5.8 and stayed at this level for the remaining incubation period. This acidification in anoxia was related to the presence of chondrocytes as cell-free conditions did not show any alteration to media pH. Oxygen levels at 2% and 5% remained steady-state whereas at 21% O_2_ levels were reduced to 12.0% at 24 h, remaining at this level throughout the rest of the incubation period. This latter finding may reflect alterations in chondrocyte physiology, such as increased oxygen consumption, when exposed to an oxygen-rich environment^24^. Despite the use of sodium dithionite, true anoxia was not achieved with mean O_2_ levels of 0.7 ± 0.4% were measured. Therefore the term anoxia used in this study meant oxygen levels <1.1%.

**Fig. 1:**
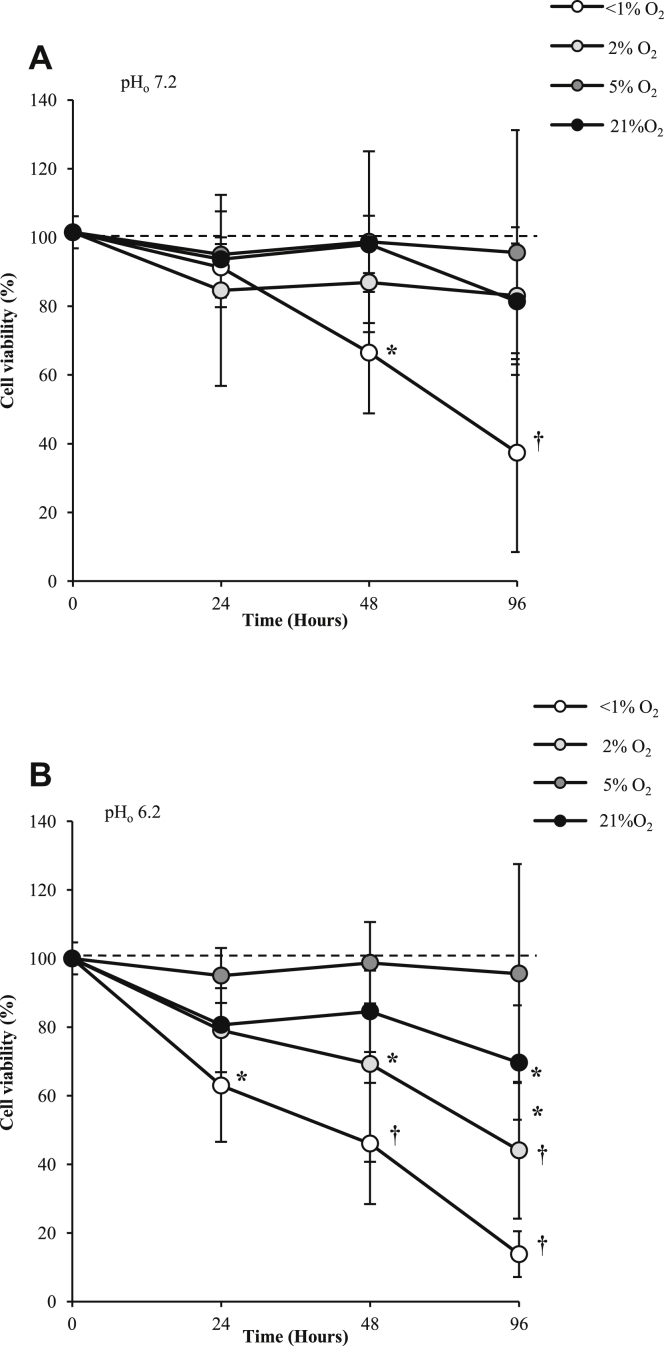
Effect of oxygen tension and pH on cell viability of human osteoarthritic articular chondrocytes over 96 h. Cell viability was determined by the trypan blue exclusion and presented as mean values compared to control (time = 0, 5% O_2_, pH 7.2). Osteoarthritic chondrocytes were cultured in three-dimensional alginate beads in <1% (anoxia), 2% (hypoxia), 5% (normoxia for chondrocytes) or 21% O_2_ (hyperoxia for chondrocytes) at pH 7.2 (normal pH) (**A**) or 6.2 (acidosis) (**B**) and cell viability was measured after 24, 48 h and 96 h. Line graphs represent means ± 95% CI, *n* = 3 donors. *P < 0.05; †*P* < 0.01.

Cell viability at pH 7.2 did not differ across all oxygen tensions after 24 h and at 48 and 96 h in 2%, 5% and 21% O_2_ but significantly reduced (*P* < 0.001) in <1% O_2_ after 96 h (Fig. 1). Cell viability was maintained in acidosis (pH 6.2) at 5% and 21% O_2_ at 24 and 48 h but reduced at 96 h in 21% O_2_ (*P* = 0.014). In hypoxia (2% O_2_) chondrocyte viability in acidotic conditions (pH 6.2) was significantly decreased after 48 and 96 h (*P* = 0.036 and 0.001, respectively), and anoxia significantly reduced cell viability at each time point (Fig. 1). The presence of IL-1β significantly reduced cell viability independent of pH at all oxygen levels. The results show significant effects of oxygen and acidosis on HOAC cell viability, amplified in the presence of the pro-inflammatory cytokine IL-1β.

### Chondrocyte GAG release

**Fig. 2:**
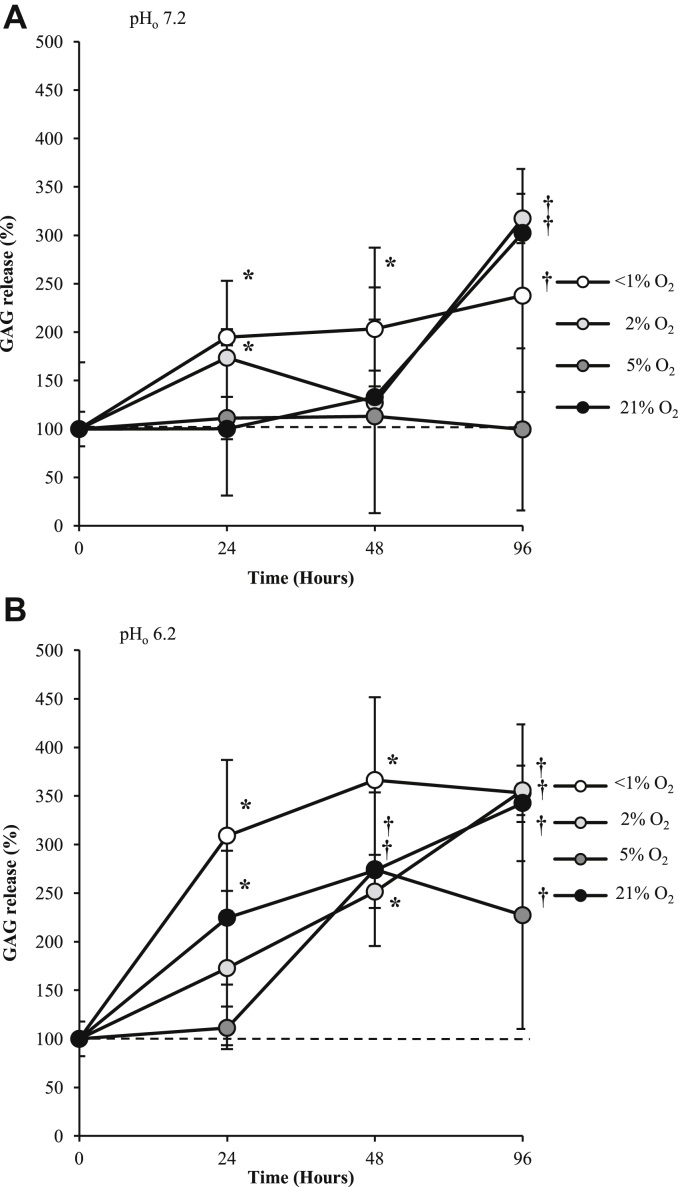
Effect of oxygen tension and pH on GAG release from human osteoarthritic articular chondrocytes over 96 h. GAG release was determined by the DMMB assay and presented as mean values compared to control (time = 0, 5% O_2_, pH 7.2). Osteoarthritic chondrocytes were cultured in three-dimensional alginate beads in <1% (anoxia), 2% (hypoxia), 5% (normoxia for chondrocytes) or 21% O_2_ (hyperoxia for chondrocytes) at pH 7.2 (normal pH) (**A**) or 6.2 (acidosis) (**B**) and GAG release into culture media was measured spent media after 24, 48 h and 96 h. Line graphs represent means ± 95% CI, *n* = 3 donors. *P < 0.05; †*P* < 0.01.

Media GAG content did not significantly alter over the 96 h incubation period at 5%O_2_ (pH 7.2) (Fig. 2). When incubated at 21% O_2_, however, significant increase in GAG release was measured after 96 h (*P* = 0.011) again, potentially reflecting response to the abnormal hyperoxic conditions relative to *in vivo* conditions. Hypoxic (2% O_2_) and anoxic (<1% O_2_) conditions significantly increased GAG release after 24 h. Acidosis (pH 6.2) also led to significant increase in GAG release in all oxygen conditions after 48 h (but only in <1% O_2_ and 21% O_2_ after 24 h). The presence of 10 ng/ml IL-1β led to a 4–5 fold increase in GAG release after 24 h in all oxygen tensions (in pH 7.2 conditions) (all *P* < 0.001).

### ATP_i_

**Fig. 3:**
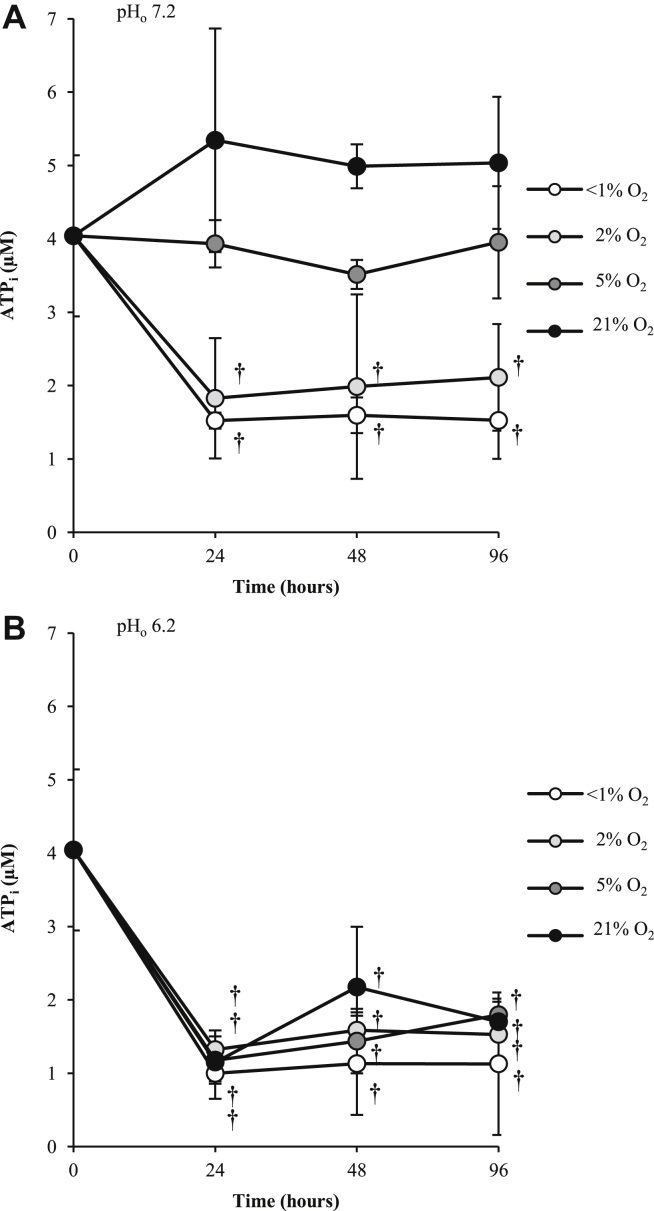
Effect of oxygen tension and pH on ATP_i_ in human osteoarthritic articular chondrocytes over 96 h. Osteoarthritic chondrocytes were cultured in three-dimensional alginate beads in <1% (anoxia), 2% (hypoxia), 5% (normoxia for chondrocytes) or 21% O_2_ (hyperoxia for chondrocytes) at pH 7.2 (normal pH) (**A**) or 6.2 (acidosis) (**B**) and ATP_i_ was determined after 24, 48 h and 96 h using the EnzyLight^™^ ATP assay kit. Line graphs represent means ± 95% CI, *n* = 3 donors. *P < 0.05; †*P* < 0.01.

ATP_i_ levels were reduced in hypoxic (2% O_2_) and anoxic (<1% O_2_) conditions after 24 h and remained reduced (Fig. 3). Interestingly, ATP production increased at 21% O_2_ compared to 5% O_2_, although this was not statistically significant. Acidosis led to significant reductions in ATP_i_ at all oxygen concentrations.

### NO levels

Nitrite/nitrate release into the media was used as a marker of NO production. Nitrite and nitrate release did not change during 96 h incubation at 5% O_2_ (pH 7.2) but nitrite release significantly increased at 21% O_2_ at 48 h, compared to control (*P* = 0.001) (Table I). Anoxia (<1% O_2_) led to reduction in measured nitrite and nitrate in all conditions, the latter likely to reflect the lack of available substrate (i.e., oxygen) for NO synthesis. Nitrate release was also increased under acidic conditions at all oxygen tensions (apart from anoxia) becoming significant in hypoxia at 48 h (*P* = 0.037).

Based on these results obtained over a 0–96 h incubation period, it appeared that 48 h represented a consistent time point for alterations in chondrocyte function in response to the hypoxia and acidosis. Therefore all further measurements were performed after 48 h incubation.

### ROS levels

**Fig. 4:**
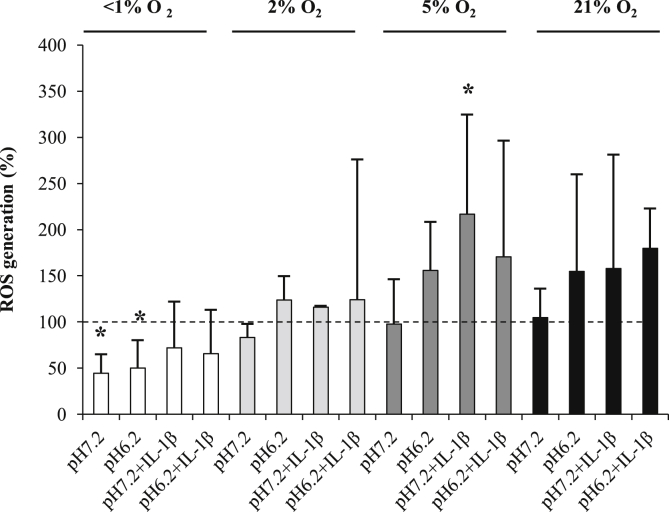
Effect of oxygen tension, pH and IL-1β on ROS levels from human osteoarthritic articular chondrocytes. ROS were measured as DCF fluorescence and presented as mean values compared to control (time = 0, 5% O_2_, pH 7.2). Osteoarthritic chondrocytes were cultured in three-dimensional alginate beads in <1% (anoxia), 2% (hypoxia), 5% (normoxia for chondrocytes) or 21% (hyperoxia for chondrocytes) O_2_ for 48 h at pH 7.2 (normal pH) or 6.2 (acidosis) in the absence or presence of IL-1β (10 ng/ml). Bar chart represents mean and 95% CI, *n* = 3 donors. *P < 0.05.

After 48 h culture, cellular ROS levels at 5% and 21% O_2_ were similar to control levels (Fig. 4). Reductions in oxygen tension led to reductions in ROS levels at pH 7.2 whereas IL-1β increased ROS levels in all oxygen conditions.

### Active MMP-13

Release of active MMP-13 into the media was not influenced by oxygen tension or pH (Supplementary material, Table S1) over the time course studied.

### Mitochondrial membrane potential (ΔΨ_m_)

**Fig. 5:**
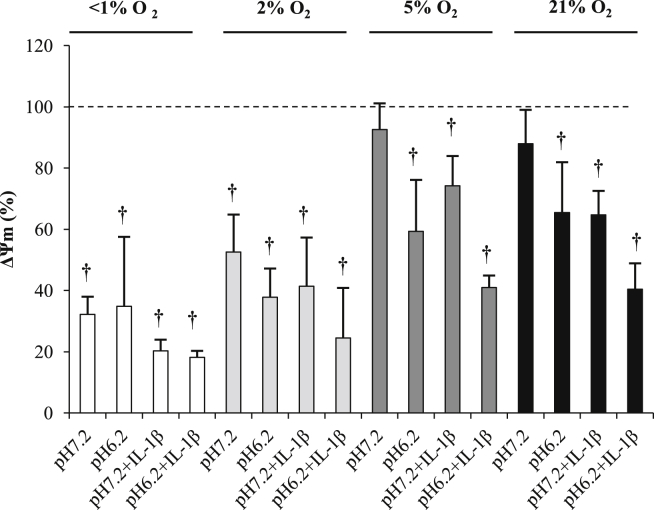
Effect of oxygen tension, pH and IL-1β on the mitochondrial membrane potential of human osteoarthritic articular chondrocytes. The mitochondrial membrane potential was measured using the fluorescent probe JC-1 and results are presented as mean values compared to control (time = 0, 5% O_2_, pH 7.2). Osteoarthritic chondrocytes were cultured in three-dimensional alginate beads in <1% (anoxia), 2% (hypoxia), 5% (normoxia for chondrocytes) or 21% (hyperoxia for chondrocytes) O_2_ for 48 h at pH 7.2 (normal pH) or 6.2 (acidosis) in the absence or presence of IL-1β (10 ng/ml). Bar chart represents mean and 95% CI, *n* = 3 donors. †*P* < 0.01.

Incubation at 5% or 21% O_2_ (pH 7.2) for 48 h did not significantly alter mitochondrial membrane potential compared to control values. Reductions in oxygen tension however, led to significantly reductions in red:green ratio indicating mitochondrial membrane depolarisation (Fig. 5). Acidosis (pH 6.2) also led to significant mitochondrial membrane depolarisation at all oxygen tensions (all *P* < 0.001), although there were no significant differences at <1% and 2% O_2_ between pH 7.2 and 6.2.

### Intracellular pH

Moderate reduction in intracellular pH occurred under anoxic conditions (<1% O_2_) (Table II). Acidosis (pH 6.2) or the presence of 10 ng/ml IL-1β did not result in further alteration in intracellular pH.

### GSG:GSSG

**Fig. 6:**
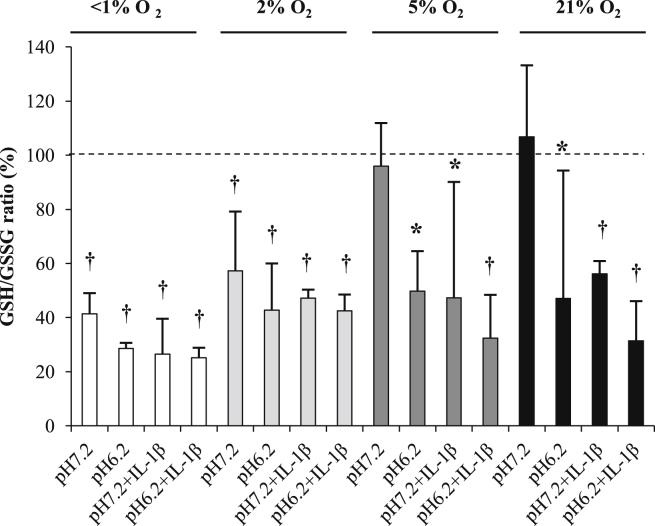
Effect of oxygen tension, pH and IL-1β on the GSH:GSSG ratio of human osteoarthritic articular chondrocytes. The GSH:GSSG ratio was measured using the luminescence based GSH/GSSG-Glo™ Assay and results are presented as mean values compared to control (time = 0, 5% O_2_, pH 7.2). Osteoarthritic chondrocytes were cultured in three-dimensional alginate beads in <1% (anoxia), 2% (hypoxia), 5% (normoxia for chondrocytes) or 21% (hyperoxia for chondrocytes) O_2_ for 48 h at pH 7.2 (normal pH) or 6.2 (acidosis) in the absence or presence of IL-1β (10 ng/ml). Bar chart represents mean ± 95% CI, *n* = 3 donors. *P < 0.05; †*P* < 0.01.

GSH:GSSG ratio decreased with reduction in oxygen tension (Fig. 6). Incubation at <1% and 2% O_2_ though led to significant reductions in GSH:GSSG ratio (both *P* < 0.001). Acidosis and the presence of IL-1β further reduced GSH:GSSG ratio in all oxygen conditions, although the effect of IL-1β was significant only at 5% and 21% O_2_.

## Discussion

This work highlights important effects of pH and oxygen on articular chondrocyte function by studying a number of relevant factors related to redox balance including NO/ROS generation, intracellular anti-oxidant levels and mitochondrial function in HOAC. Here we show that HOACs are sensitive to acidosis and very low levels of oxygen, conditions occurring in the diseased joint^6–9^, and exacerbated by the pro-inflammatory cytokine IL-1β. These findings are important considerations when studying osteoarthritis and emphasise that intracellular oxidant systems are likely to be key players in maintaining chondrocyte viability. Finally this work underlines the effect of culture conditions on chondrocyte activity and that studying chondrocyte biology at non-physiological levels of oxygen may be inappropriate.

For the purposes of this study we used human articular chondrocytes isolated from osteoarthritic joints since the primary aim was to evaluate the response of diseased tissue to alterations in the extracellular environment (namely pH and oxygen). Comparison to non-diseased tissue would have been advantageous to determine differences of the disease to cell responses to alterations in the physicochemical environment but this was not achievable in this project. Direct comparison can often be complicated due to inherent heterogeneity of both normal and diseased tissue and the difficulty in obtaining age-matched controls.

This study adopted a primary 3-D alginate culture system as opposed to using monolayer culture. It is widely accepted that chondrocyte de-differentiation occurs in monolayer culture with loss of collagen type II expression and that a three-dimensional culture environment allows for maintenance of a chondrocytic phenotype^23,25^. Although initially we employed monolayer culture to expand our cell numbers (a necessary step needed to perform the number of analyses in this study), subsequent examination following 3-D alginate cell culture showed an articular chondrocyte phenotype still being expressed (Fig. S1). Despite this, we cannot fully rule out that de-differentiation may have occurred during culture, though our findings point to the maintenance of an articular phenotype prior to and after the experimental period. It is therefore crucial to consider the effects of culture conditions on chondrocyte activity at every level.

The effects of extracellular pH on chondrocyte function have received less attention than oxygen. In a recent study^11^, extracellular pH appeared to have a stronger effect than oxygen on chondrocyte function (although it is to be noted that 5% O_2_ was the lowest oxygen tension evaluated in that study). Cartilage matrix synthesis has a bimodal response to alterations in extracellular pH with optimal synthesis occurring between pH 7.0–7.2 and synovial fluid acidosis occurs in osteoarthritis and rheumatoid arthritis suggesting pH may have an important role in maintaining cartilage integrity^5,26–28^.

In our study, acidosis was an important regulator of chondrocyte function and redox balance. Incubation at pH 6.2 reduced chondrocyte viability and ATP_i_ levels and increased GAG release into the media, particularly notable at low oxygen tensions (2% and <1% O_2_). In addition, acidosis had a significant effect by increasing nitrite and nitrate release in all oxygen tensions (apart from <1% O_2_). Increased production of NO has been reported in other cells in response to extracellular acidification^14^ and NO interaction with hydroxyl radicals to produce peroxynitrite *via* the Fenton reaction is important regulator of matrix synthesis and chondrocyte apoptosis in cartilage^29,30^. Here, we show increased cellular ROS levels in response to extracellular acidosis and although we did not measure changes in peroxynitrite levels and protein nitration, these may have provided an additional indicator of redox imbalance and cellular damage. NO also potentiates acid-sensing ion channel (ASIC) mediated neuronal cell death in low pH conditions^31^ and blocking ASIC has been recently shown to reduce chondrocyte apoptosis in an acid-induced (pH 6.0) apoptotic model^32^. In this study, we demonstrated mitochondrial membrane potential depolarisation in acidic conditions (at all oxygen levels). Since ATP production by oxidative phosphorylation is linked to mitochondrial membrane potential, depolarisation in acidosis and reduced oxygen tensions may be responsible for reduced ATP_i_ levels, although complicated interactions between ATP_i_ regulation (production, transport, store and release) exist on numerous levels in the cell^33^. In addition, regulation of glycolytically-derived ATP in chondrocytes may be pH and oxygen-sensitive^34^, possibly linked through ROS levels^35^. Rong and co-workers^36^ recently demonstrated that partial restoration of mitochondrial membrane depolarisation in response to extracellular acidosis led to reduction in apoptotic rate in rat articular chondrocytes and with our work highlights a potential therapeutic target.

We also measured significant reductions in cellular GSH:GSSG ratio in HOAC following acidosis. Reductions in GSH have been reported in other cells, such as hepatocytes^14^ in response to acidification and NO may inhibit GSH reductase activity thereby modifying GSH content^37^. Additionally, with the findings that GSH depletion results in the decrease in activity of Na^+^/H^+^ exchanger^38^, leading to intracellular acidification, this could provide a potential mechanism to link extracellular acidosis, mitochondrial membrane potential, NO and GSH content with intracellular acidification and cell viability in chondrocytes and indicates that further work is required in this area.

The importance of oxygen as a regulator of chondrocyte activity has gained increasing recognition^13^. *In vivo* levels of oxygen in the joint are relatively hypoxic compared to other tissues^5^ with diffusion gradients down superficial to deep cartilage zones existing^3^. Chondrocyte matrix synthesis has been shown to be oxygen-sensitive with optimal matrix synthesis occurring between 5% and 10%^39,40^. Although chondrocytes can still survive and produce matrix in very low oxygen tensions^9^, other work shows increased apoptosis at 1% and 21% compared to 5% O_2_^41^.

The effects of very low oxygen tensions on chondrocyte function can be difficult to determine in the literature, partly due to the variable use of the terms hypoxia and anoxia in cartilage studies. In this study we defined <1% O_2_ as anoxia (since true anoxia could not be achieved and we measured an average of 0.7% O_2_ in our anoxic culture conditions) and hypoxia as 2% O_2_ since “normoxia” for chondrocytes *in vivo* is around 5% O_2_. Ambient values of 21% O_2_ may therefore represent abnormally high levels of oxygen and lead to potentially inappropriate chondrocyte responses^24,42–44^. In our study, 5% O_2_ appeared to result in the least deviation from control values and some parameters, such as GAG and nitrite/nitrate levels in media, were increased over time after culturing at 21% O_2_.

Hypoxia and anoxia resulted in reductions in cell viability and increase in GAG release, accentuated in acidic conditions and also in the presence of IL-1β. Very low oxygen levels led to intracellular acidification, previously reported in normal equine articular chondrocytes^12^. Nitrite and nitrate release into media was modestly increased in hypoxic conditions but reduced in anoxia. The latter may reflect the lack of availability in oxygen as a substrate in NO synthesis. Mitochondrial membrane potential and ROS levels however were decreased in hypoxia and anoxia. Since the main source of ROS generation in chondrocytes appeared to be mitochondrially derived, and since ROS generation is intimately related to mitochondrial membrane potential it is likely that the ROS reductions observed are due to mitochondrial membrane depolarisation under low or very low oxygen conditions. We have documented this link previously in normal equine articular chondrocytes under short-term hypoxic conditions^45^ and therefore this study extends these observations in longer-term hypoxia in HOAC.

Similar to acidosis, reduction in oxygen levels led to significant reduction in GSH:GSSG. The ratio of reduced (GSH) to oxidised glutathione (GSSG) is an indicator of cellular redox status and dependent on activities of anti-oxidant enzymes such as glutathione peroxidase^46^. The effects of hypoxia on GSH:GSSG has been documented in other tissues, and is related to age-related and/or disease. For example, chronic hypoxia reduces GSH:GSSG in brain vessels and may potentiate oxidative imbalance seen in age-related neurodegeneration^15^. Additionally, reduction in glutathione peroxidise (GPx) activity following chronic hypoxia has been documented in hepatocytes leading to lower GSH synthetic rates^47^. Reduction in GPx and other anti-oxidant enzymes, such as superoxide dismutase 2 (SOD2) occurs in osteoarthritis^16,17^ and could be linked to the further reduction in oxygen levels occurring in osteoarthritis (OA). Moreover, IL-1β disrupts the cellular anti-oxidant enzymes in chondrocytes, including GPx and SOD as a potential cause of oxidative stress^48^.

This work shows that oxygen and pH are important factors in relation to elements of the cellular redox system in osteoarthritic chondrocytes. Further work detailing these interactions particularly involving the anti-oxidant enzyme systems such as GPx and SOD under these conditions is therefore highly relevant.

## Author contributions

JC participated in study design, majority of the experimental work and analysis and interpretation of the data. RW undertook some experimental work and data analysis. RM and PC participated in study design and interpretation of data. PM participated in study design, some experimental work, analysis and interpretation of data. All authors contributed to, read and approved the final manuscript.

## Role of the funding source

JC was supported by BBSRC and MSD.

## Conflict of interest

The authors have no conflicts of interest to report.

## Figures and Tables

**Table I:** Nitrite and nitrate release (μM) from human osteoarthritic articular chondrocytes after 24, 48 and 96 h incubation in <1%, 2%, 5% or 21% O_2_ in pH 7.2 or 6.2. Results represent mean (95% CI) of three individual donors vs control (5% O_2_, pH 7.2, 0 h). Bold indicates *P* value <0.05

		pH 7.2		pH 6.2	
		Mean (95% CI)	*P*-value	Mean (95% CI)	*P*-value
Nitrite (μM)					
5% O_2_	0 h	0.20 (0.13, 0.26)	–	–	–
21% O_2_	24 h	0.17 (0.09, 0.25)	0.81	0.27 (0.20, 0.35)	0.63
	48 h	0.43 (0.29, 0.58)	**0.001**	0.32 (0.16, 0.47)	0.32
	96 h	0.30 (0.21, 0.38)	0.12	0.31 (0.01, 0.61)	0.36
5% O_2_	24 h	0.14 (0.07, 0.21)	0.45	0.27 (0.17, 0.34)	0.66
	48 h	0.23 (0.11, 0.34)	0.87	0.35 (0.23, 0.46)	0.21
	96 h	0.21 (0.05, 0.36)	0.99	0.24 (−0.09, 0.57)	0.92
2% O_2_	24 h	0.16 (0.02, 0.30)	0.91	0.26 (0.05, 0.48)	0.85
	48 h	0.25 (0.18, 0.32)	0.83	0.41 (0.17, 0.65)	0.15
	96 h	0.25 (−0.07, 0.57)	0.82	0.34 (0.00, 0.67)	0.41
<1% O_2_	24 h	0.05 (0.03, 0.07)	**0.002**	0.03 (0.01, 0.06)	**0.001**
	48 h	0.07 (−0.01, 0.15)	**0.004**	0.05 (−0.03, 0.12)	**0.001**
	96 h	0.09 (0.02, 0.16)	**0.012**	0.09 (0.03, 0.16)	**0.011**
Nitrate (μM)					
5% O_2_	0 h	18.6 (17.7, 19.5)	–	–	–
21% O_2_	24 h	17.1 (5.3, 29.0)	0.93	20.5 (10.9, 30.1)	0.98
	48 h	25.7 (22.0, 29.5)	0.57	26.0 (22.4, 29.6)	0.47
	96 h	19.6 (7.0, 32.3)	1.00	22.2 (0.0, 44.5)	0.86
5% O_2_	24 h	21.0 (11.9, 30.1)	0.90	23.3 (13.2, 33.3)	0.66
	48 h	21.0 (16.3, 25.7)	0.91	20.2 (24.4, 34.0)	0.12
	96 h	18.8 (3.6, 33.9)	1.00	24.0 (6.2, 41.8)	0.56
2% O_2_	24 h	19.0 (11.0, 27.1)	1.00	31.7 (18.9, 44.6)	**0.037**
	48 h	26.3 (22.0, 30.5)	0.39	35.8 (26.7, 44.9)	**0.009**
	96 h	21.9 (−0.3, 44.2)	0.88	29.7 (18.2, 41.2)	0.08
<1% O_2_	24 h	7.7 (5.6, 9.9)	**<0.001**	8.29 (7.70, 8.88)	**<0.001**
	48 h	7.4 (2.2, 12.5)	**<0.001**	7.7 (4.4, 11.0)	**<0.001**
	96 h	4.3 (4.0, 4.6)	**<0.001**	5.2 (3.6, 6.7)	**<0.001**

**Table II:** Intracellular pH of human osteoarthritic articular chondrocytes incubated in <1%, 2%, 5% or 21% O_2_ for 48 h in pH 7.2 or 6.2 in the presence (+) or absence (−) of 10 ng/ml IL-1β. Results represent mean (95% CI) of three individual donors vs control (5% O_2_, pH 7.2, 0 h). Bold indicates *P* value <0.05

	pH	IL-1β (10 ng/ml)	Mean (95% CI)	*P*-value
21% O_2_	7.2	–	7.32 (7.22, 7.42)	0.10
		+	7.19 (7.16, 7.22)	0.49
	6.2	–	7.16 (7.06, 7.26)	0.55
		+	7.17 (7.12, 7.22)	0.61
5% O_2_	7.2	–	7.42 (7.21, 7.63)	0.10
		+	7.32 (7.18, 7.40)	0.12
	6.2	–	7.21 (7.05, 7.36)	0.17
		+	7.14 (7.11, 7.19)	0.80
2% O_2_	7.2	–	7.25 (7.02, 7.48)	0.32
		+	7.30 (7.01, 7.59)	0.12
	6.2	–	7.18 (7.09, 7.27)	0.81
		+	7.24 (6.89, 7.59)	0.28
<1% O_2_	7.2	–	6.85 (6.80, 6.90)	**0.009**
		+	6.85 (6.71, 6.99)	**0.01**
	6.2	–	6.89 (6.79,6.99)	**0.001**
		+	6.80 (6.61, 6.99)	**0.005**

## Appendix A Supplementary data

Fig. S1 Western blot analysis for COL2A1, COMP and SOX9 expression in human osteoarthritic chondrocytes from three individual donors following 14-day incubation period in 3-D alginate beads (**A**) prior to (*t* = 0) and (**B**) following 96-hour experimental period (<1%, 2%, 5% or 21% O_2_ at pH 7.2 or 6.2). **C–E** show representative full Western blots at *t* = 0 (**C**) and following 96-hour experimental period at pH 7.2 (**D**) and 6.2 (**E**) at <1%, 2%, 5% or 21% O_2_. Alpha tubulin was used a loading control.
