# Supplementary material for: Oxygen and pH-sensitivity of human osteoarthritic chondrocytes in 3-D alginate bead culture system
Source: Osteoarthritis Cartilage. 2013 Nov;21(11):1790–8. doi: 10.1016/j.joca.2013.06.028 (PMC3807787; doi:10.1016/j.joca.2013.06.028)
Supplement: Supplementary file 1 [file mmc1.docx]

Table S1. MMP-13 (ng/ml) release from human osteoarthritic articular chondrocytes incubated in <1%, 2%, 5% or 21% O_2_ in pH7.2 or 6.2 after 24, 48 and 96 hours. Results represent mean (95% CI) of three individual donors.

| Oxygen tension | pH_o_ 7.2 | | | pH_o_ 6.2 | | |
| --- | --- | --- | --- | --- | --- | --- |
|  | 24 hrs | 48 hrs | 96 hrs | 24 hrs | 48 hrs | 96 hrs |
| 21% O_2_ | 0.12 (0.11, 0.13) | 0.14 (0.13, 0.15) | 0.18 (0.14, 0.22) | 0.16 (0.14, 0.19) | 0.19 (0.08, 0.31) | 0.24 (0.19, 0.29) |
| 5% O_2_ | 0.11 (0.07, 0.15) | 0.17 (0.11, 0.13) | 0.14 (0.13, 0.15) | 0.20 (0.16, 0.24) | 0.22 (0.16, 0.28) | 0.25 (0.20, 0.31) |
| 2% O_2_ | 0.10 (0.06, 0.14) | 0.20 (0.10, 0.30) | 0.20 (0.16, 0.24) | 0.18 (0.13, 0.23) | 0.23 (0.18, 0.29) | 0.23 (0.15, 0.30) |
| <1% O_2_ | 0.13 (0.09, 0.16) | 0.17 (0.07, 0.28) | 0.21 (0.19, 0.21) | 0.19 (0.15, 0.23) | 0.22 (0.15, 0.29) | 0.23 (0.22, 0.24) |
