# Supplementary figures and images for: Oxygen and pH-sensitivity of human osteoarthritic chondrocytes in 3-D alginate bead culture system
Source: Osteoarthritis Cartilage. 2013 Nov;21(11):1790–8. doi: 10.1016/j.joca.2013.06.028 (PMC3807787; doi:10.1016/j.joca.2013.06.028)

A

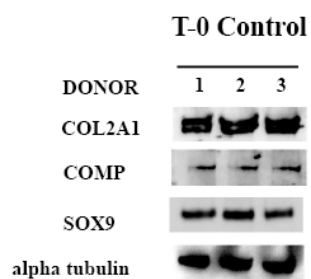

B

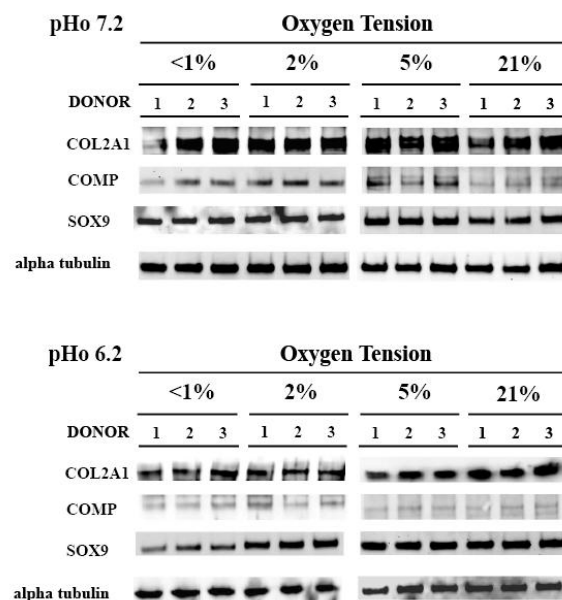

C

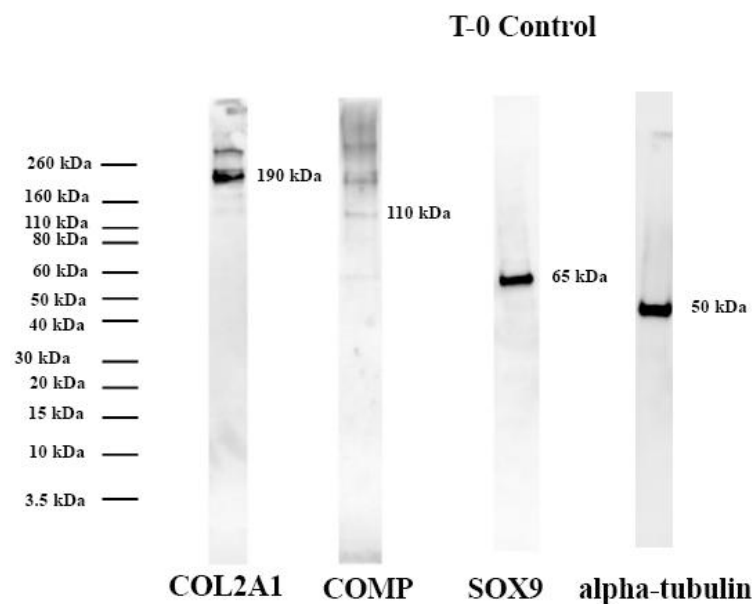

D

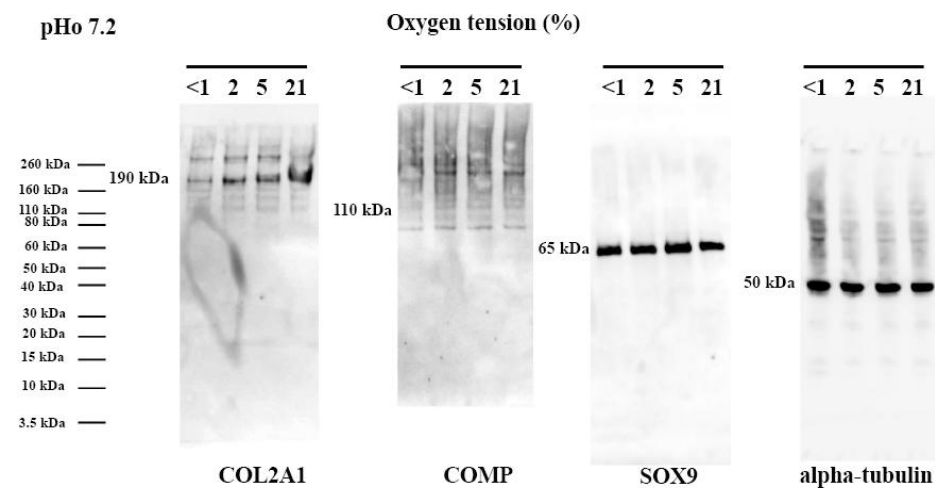

E

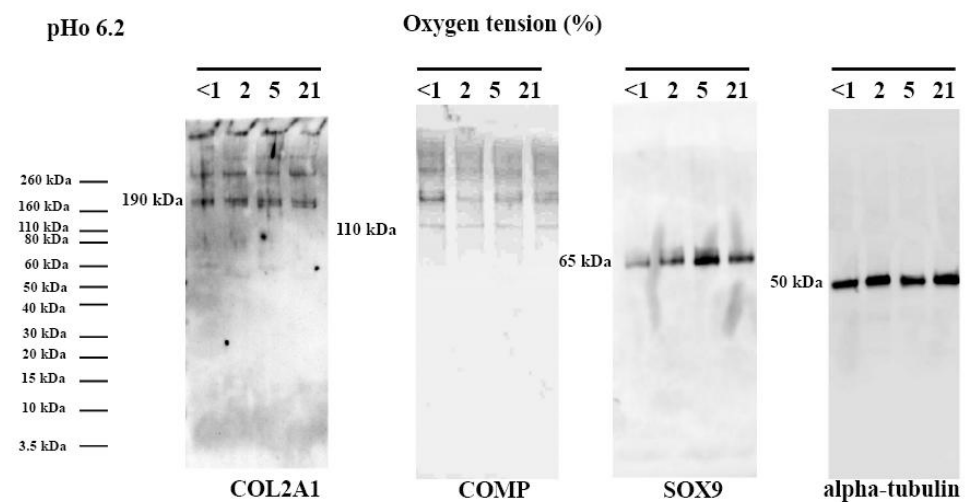

Supplement: Fig. S1 — Western blot analysis for COL2A1, COMP and SOX9 expression in human osteoarthritic chondrocytes from three individual donors following 14-day incubation period in 3-D alginate beads (A) prior to (t = 0) and (B) following 96-hour experimental period (<1%, 2%, 5% or 21% O2 at pH 7.2 or 6.2). C–E show representative full Western blots at t = 0 (C) and following 96-hour experimental period at pH 7.2 (D) and 6.2 (E) at <1%, 2%, 5% or 21% O2. Alpha tubulin was used a loading control. [file mmc2.pdf]
